# Relevance of Pharmacogenomics and Multidisciplinary Management in a Young-Elderly Patient With *KRAS* Mutant Colorectal Cancer Treated With First-Line Aflibercept-Containing Chemotherapy

**DOI:** 10.3389/fonc.2020.01155

**Published:** 2020-08-04

**Authors:** Gemma Bruera, Antonio D'Andrilli, Maurizio Simmaco, Stefano Guadagni, Erino Angelo Rendina, Enrico Ricevuto

**Affiliations:** ^1^Oncology Territorial Care, S. Salvatore Hospital, Oncology Network ASL1 Abruzzo, University of L'Aquila, L'Aquila, Italy; ^2^Department of Biotechnological and Applied Clinical Sciences, University of L'Aquila, L'Aquila, Italy; ^3^Thoracic Surgery, S. Andrea Hospital, Faculty of Medicine and Psychology, University La Sapienza, Rome, Italy; ^4^Advanced Molecular Diagnostics, S. Andrea Hospital, Rome, Italy; ^5^Universitary General Surgery, S. Salvatore Hospital, Oncology Network ASL1 Abruzzo, University of L'Aquila, L'Aquila, Italy

**Keywords:** aflibercept/chemotherapy, case report, young-elderly unfit MCRC, multidisciplinary management, pharmacogenomic analyses

## Abstract

**Introduction:** Intensive oncological treatment integrated with resection of metastases raised the clinical outcome of metastatic colorectal cancer (MCRC). In clinical practice, complex evaluation of clinical (age, performance status, comorbidities), and biological (tumoral genotype, pharmacogenomic) parameters addresses tailored, personalized multidisciplinary treatment strategies. Patients with MCRC unsuitable for first-line intensive medical treatments are prevalent and showed worse clinical outcome. After progression to oxaliplatin-based chemotherapy, aflibercept/FOLFIRI significantly improved clinical outcome, even if no survival benefit was reported in adjuvant fast relapsers by aflibercept addition. The case reported a young-elderly (yE) patient with *KRAS* mutant colorectal cancer rapidly progressing to adjuvant chemotherapy, unfit owing to comorbidities, with multiple pharmacogenomic alterations, who gained long-term survival in clinical practice by multidisciplinary treatment strategy consisting of first-line and re-introduction of aflibercept-containing chemotherapy and two-stage lung metastasectomies.

**Case presentation:** A 71-years-old yE patient, unfit for intensive oncological treatments owing to Cumulative Illness Rating Scale (CIRS) stage secondary, affected by *KRAS* c.35 G>T mutant colorectal cancer, rapidly progressing with lung metastases after adjuvant XelOx chemotherapy, reached long-term survival 66 months with no evidence of disease after first-line and re-introduction of tailored, modulated aflibercept (4 mg/kg) d1,15-irinotecan (120 mg/m^2^) d1,15-5-fluorouracil (750 mg/m^2^/day) dd1–4, 15–18; and secondary radical bilateral two-stage lung metastasectomies. Safety profile was characterized by limiting toxicity syndrome at multiple sites (LTS-ms), requiring 5-fluorouracil discontinuation and aflibercept reduction (2 mg/kg), because of G2 hand-foot syndrome (HFS) for >2 weeks, and G3 hypertension. Pharmacogenomic analyses revealed multiple alterations of fluoropyrimidine and irinotecan metabolism: severe deficiency of fluorouracil degradation rate (FUDR), single nucleotide polymorphisms of UGT1A1^*^28 variable number of tandem repeats (VNTR) 7R/7R homozygote, *ABCB1* c.C3435T, c.C1236T, *MTHFR* c.C667T homozygote, *DPYD* c.A166G, *TSER* 28bp VNTR 2R/3R heterozygote.

**Conclusions:** In clinical practice, a complex management evaluating clinical parameters and *RAS*/*BRAF* genotype characterizing an individual patient with MCRC, particularly elderly and/or unfit owing to comorbidities, is required to properly address tailored, multidisciplinary medical and surgical treatment strategies, integrated with careful monitoring of superimposing toxicity syndromes, also related to pharmacogenomic alterations, to gain optimal activity, and long-term efficacy.

## Introduction

Activity of intensive medical treatment integrated with surgical resection of metastases raised the effectiveness of clinical outcome of patients with metastatic colorectal cancer (MCRC). We previously demonstrated that first-line intensive FIr-B/FOx triplet chemotherapy plus bevacizumab reached an objective response rate (ORR) of 82%, median progression-free survival (PFS) of 12 months, and overall survival (OS) of 28 months (1, 2). High activity correlated with 26% secondary liver resections and 15% pathologic complete response (CR) (3). Integrated multidisciplinary treatments significantly improved clinical outcome of liver-limited patients (PFS 17 months, OS 44 months), compared with other/multiple metastatic sites (O/MM) (3), not significantly affected by *KRAS*/*NRAS*/*BRAF* genotype (4). In non-elderly *RAS* wild-type patients, FIr-C/FOx-C triplet chemotherapy plus cetuximab was highly active and tolerable at recommended doses, with PFS 12 months, confirming that intensive first-line regimens increase efficacy, also by increasing secondary resection of liver metastases (5). In patients progressing after oxaliplatin-based first-line treatment, aflibercept addition to FOLFIRI significantly improved OS to 13.5 months, PFS to 6.9 months, and ORR to 19.8% (6). Patients fast relapsing to adjuvant chemotherapy showed poorer efficacy and no survival benefit by aflibercept addition (OS 10.4 vs. 9.6 months) (7, 8).

In clinical practice, a complex evaluation of clinical (age, performance status, comorbidity status) and biological (*KRAS/NRAS/BRAF* genotype) parameters addresses tailored, multidisciplinary treatment strategies (9). Patients unsuitable for first-line FIr-B/FOx regimen due to old-elderly status (≥75 years), performance status (PS) ≥2, and/or comorbidities were prevalent, mostly elderly, PS 1-2, CIRS stage intermediate/secondary (7), O/MM (9, 10); they were treated with tailored triplet or doublet first-line treatments, and showed worse clinical outcome (11). Thus, in unfit patients, it is challenging to select the proper, modulated treatment regimen, weighing expected efficacy with safety profile (9). To this aim, we recently added the evaluation of toxicity syndromes (TS), specifically limiting TS (LTS), evaluating the spectrum of limiting and non-limiting toxicities observed in the individual patient (12). Evaluation of LTS integrated with pharmacogenomic analysis of fluorouracil and irinotecan metabolism can be useful to personalize treatment schedule and doses (5).

We reported an experience in clinical practice of multidisciplinary management of a yE patient with *KRAS* mutant colorectal cancer rapidly progressing to adjuvant chemotherapy, unfit for intensive medical treatment, owing to yE and comorbidities, who reached long-term OS with no evidence of disease after first-line and re-introduction of aflibercept-containing chemotherapy integrated with secondary, bilateral, two-stage lung metastasectomies.

## Clinical Case Presentation

A 71-years-old man, with secondary CIRS stage (10), resulting from hypertensive cardiopathy, dyslipidemia, diabetes on treatment, and positive cancer family history (mother with unspecified cancer at 75 years, brother with unspecified bone tumor, son with fibrosarcoma diagnosed at 8 months, relapsed at 17 years and surgically treated), underwent right colectomy for an ulcerative, stenotic lesion of 3 × 2.5 cm, microscopically defined as moderately differentiated adenocarcinoma with 5% mucinous component, infiltrating colic wall and pericolic fat, infiltrating pattern, poor lymphocytic infiltration, mesenteric tumoral nodes, negative resection margins, four out of 30 metastatic regional lymph nodes, stage pT3 pN2a, *KRAS* mutant c. 35 G>T genotype. Preoperative CT scan and postoperative PET did not show metastatic disease. Because of the elderly status and secondary CIRS stage, the patient underwent adjuvant chemotherapy according to the following schedule: oxaliplatin (120 mg/m^2^) d1, capecitabine (825 mg/m^2^ bid) d1–14, cycles repeated every 21 days, for six cycles. Safety profile was characterized by LTS-ms, specifically G2 HFS associated with G2 anemia (1, 4, 12, 13). At disease-free survival (DFS) 10 months and disease-free interval (DFI) 4 months after completion of adjuvant chemotherapy, CT scan showed bilateral lung metastases at left antero-basal (8 mm), right inferior (7 mm), and posterior–superior lobe (3 mm), confirmed by PET. CEA, CA19.9 tumor markers were negative.

**Figure 1:**
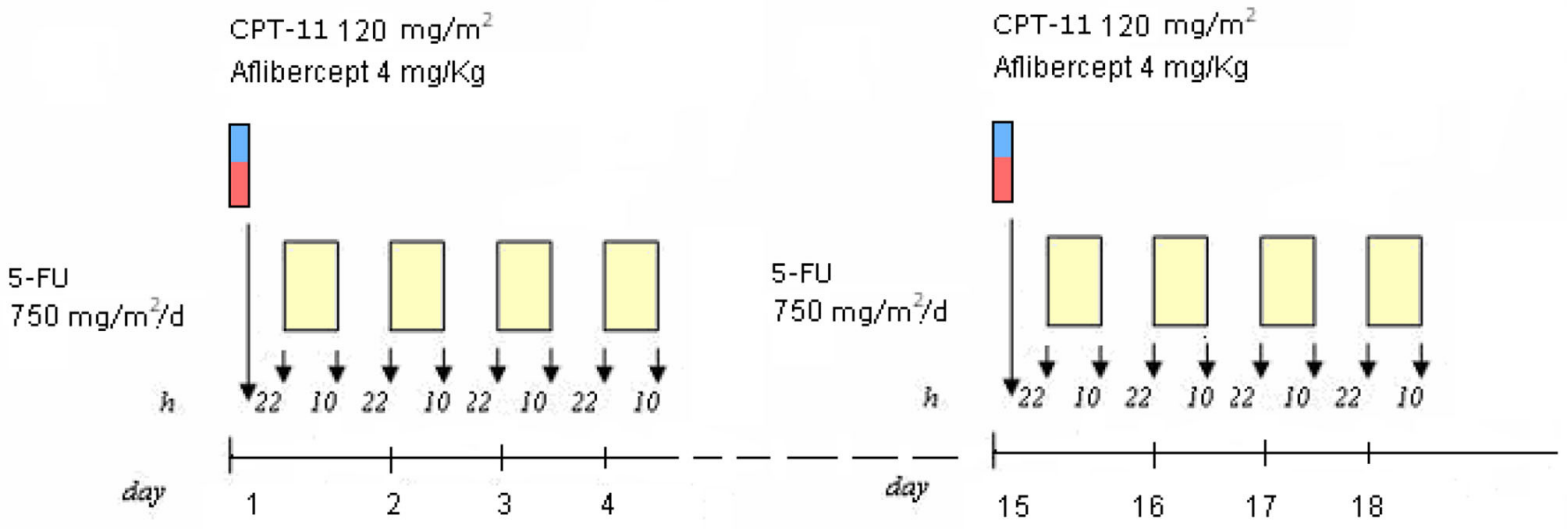
Planned treatment schedule.

**Figure 2:**
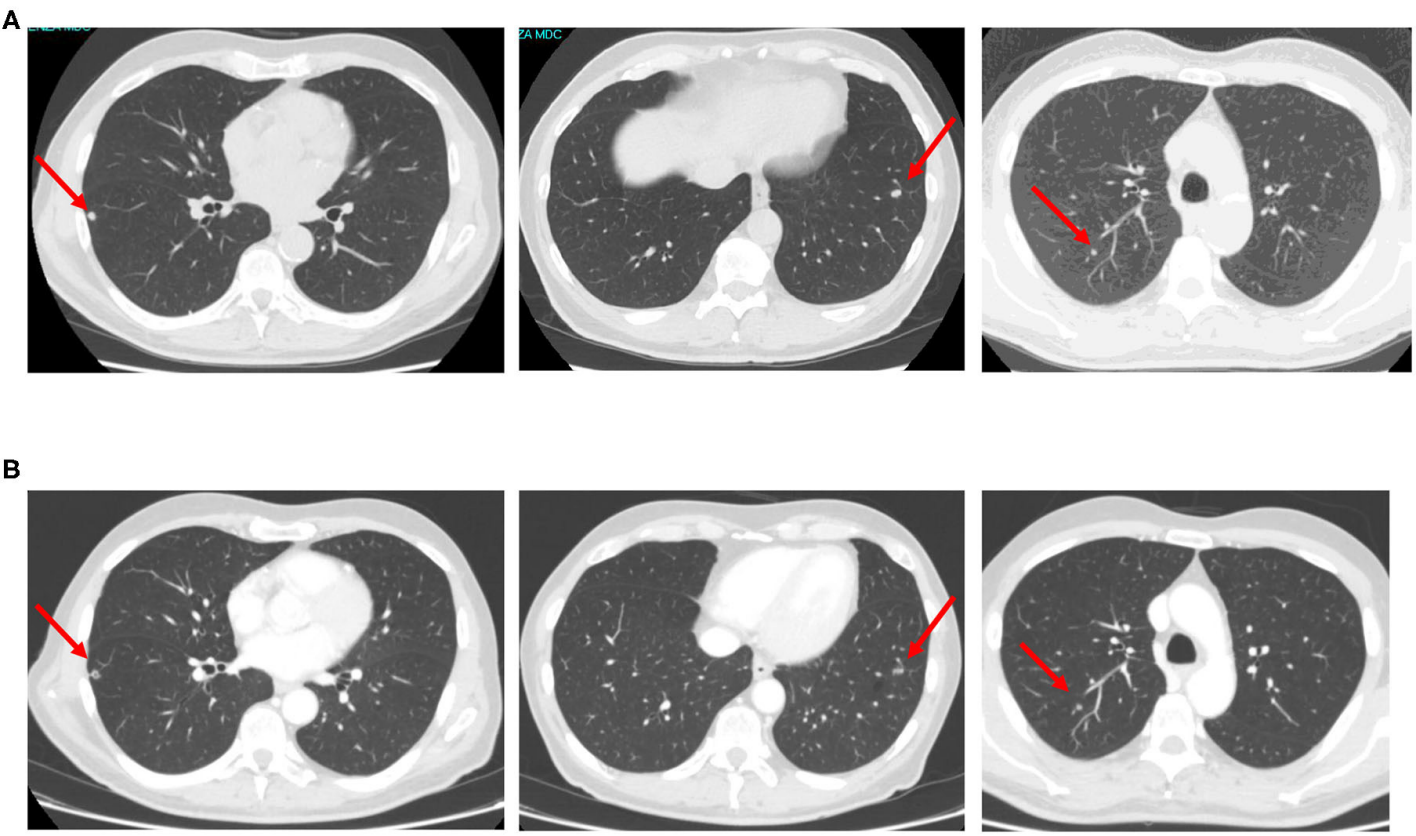
CT scan images showing bilateral lung metastases early relapsed after adjuvant chemotherapy **(A)** and re-evaluated after first-line aflibercept/chemotherapy **(B)**.

**Figure 3:**
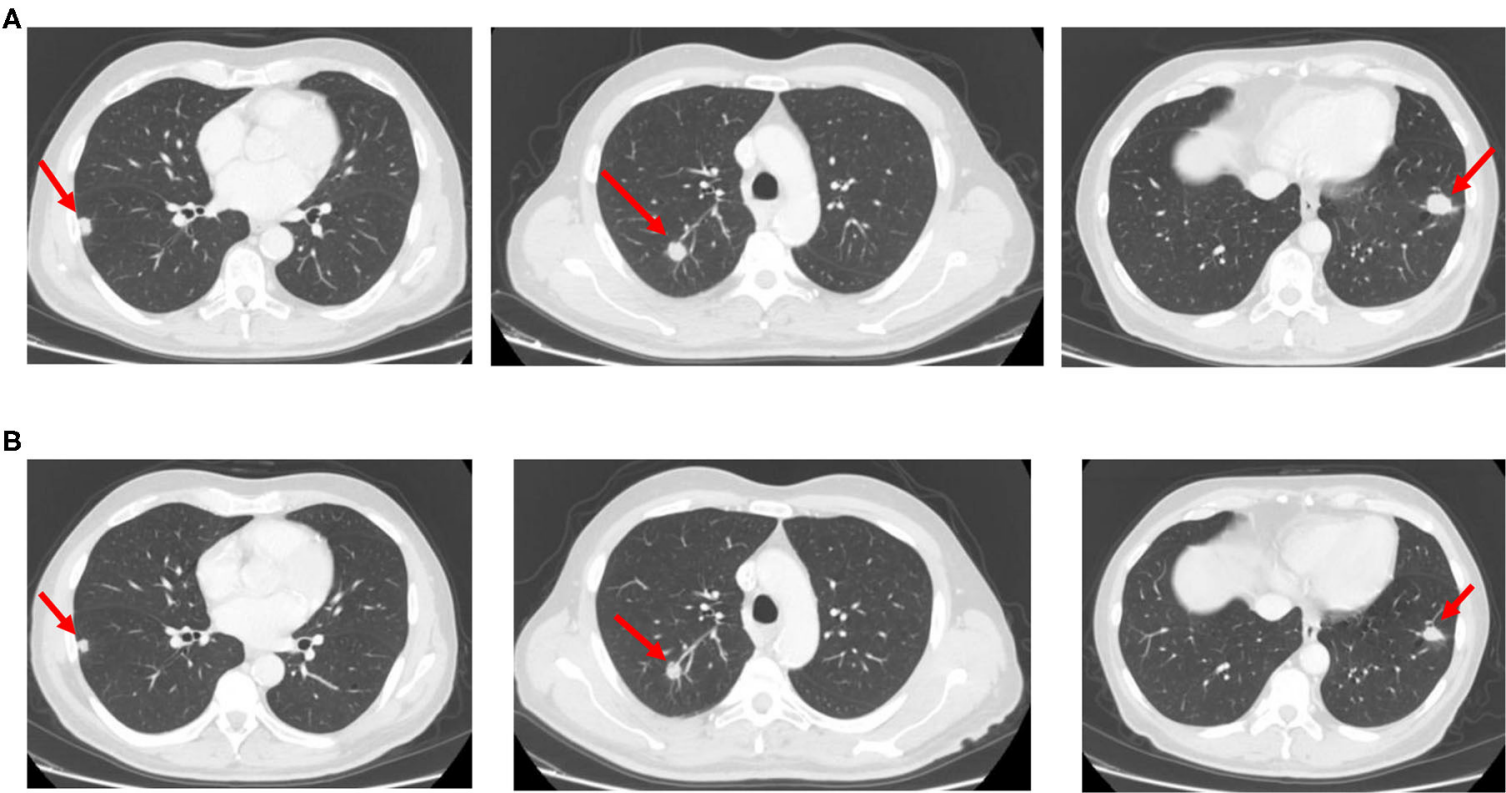
CT scan images showing bilateral lung metastases relapsed after first-line chemotherapy **(A)** and re-evaluated after aflibercept/chemotherapy re-introduction **(B)**.

The yE patient with secondary CIRS stage, *KRAS* mutant c.35 G>T MCRC rapidly relapsing after adjuvant XelOx chemotherapy, previously experiencing LTS-ms, underwent first-line modulated treatment according to the following schedule: aflibercept (4 mg/kg) d1,15-irinotecan (120 mg/m^2^) d1,15-5-fluorouracil (750 mg/m^2^/day) dd1–4, 15–18, cycles repeated every 28 days (Figure 1), based on previously reported doublet fluorouracil/irinotecan schedule (14). Received dose intensities were 100% of planned. Safety profile was characterized by LTS-ms: G2 HFS for >2 weeks, G3 alopecia, G2 rhinitis, G1 hypertension, mucositis, epistaxis, asthenia, dysphonia, diarrhea, and bilirubin increase. Pharmacogenomic analyses showed multiple alterations involving fluoropyrimidine and irinotecan metabolism: severe deficiency of fluorouracil degradation rate (FUDR) 0.51 ng/min/mil.cell (severe deficiency cut-off <0.68 ng/min/mil.cell), single nucleotide polymorphisms (SNPs) of UGT1A1^*^28 homozygote VNTR 7R/7R, *ABCB1* heterozygote c.C3435T, c.C1236T, *MTHFR* homozygote c.C667T, *DPYD* heterozygote c.A166G, *TSER* 28bp heterozygote VNTR 2R/3R. First evaluation of response by CT scan showed partial response (PR) of secondary lung metastases with intra-lesion necrosis (Figure 2). Because of limiting HFS at 5-FU dose intensity 1,500 mg/m^2^/week and pharmacogenomic alterations, specifically reduced FUDR, 5-fluorouracil was discontinued, and further aflibercept/irinotecan three cycles were planned. CT scan after six cycles confirmed PR. Patient underwent further three aflibercept/irinotecan cycles. Safety profile was characterized by G3 hypertension, G3 alopecia, G1 HFS, mucositis, rhinitis, epistaxis, asthenia, and dysphonia. After nine cycles, with persistent PR, treatment was discontinued owing to limiting G3 hypertension. At PFS 16 months and PFI 5 months off-treatment, CT scan showed progression of lung metastases. Re-challenge of the same regimen was proposed for three cycles, according to the following schedule, because of previous limiting G3 hypertension: aflibercept 2 mg/kg d1, 15-irinotecan 120 mg/m^2^ d1, 15, every 28 days. Received dose intensities were 100%. Safety profile was characterized by G3 alopecia, G2 hypertension, G1 mucositis, rhinitis, epistaxis, asthenia, and dysphonia. First evaluation of response by CT scan showed 50% PR of secondary lung nodules (Figure 3). Treatment was planned for further three cycles. Tolerability was characterized by G3 alopecia, G2 hypertension, G1 mucositis, rhinitis, epistaxis, asthenia, and dysphonia. PET scan confirmed metabolic PR at PFS 7 months.

Because of long-term control of bilateral lung metastastes during aflibercept/irinotecan first-line and re-challenge treatments, low disease burden, even if bilateral lung metastases, multidisciplinary treatment strategy was shared with thoracic surgeons, and bilateral lung resections were planned. The patient underwent atypical resection of right dorsal segment of superior lobe and apical segment of inferior lobe; metastatic lesions of 1.4 cm and 0.8 cm of mucinous colon adenocarcinoma were diagnosed. Nine weeks after, the patient underwent second-stage atypical resection of inferior lobe. Histological examination confirmed a sub-pleural partially necrotic lesion of 2.4 cm, with mucinous features of colon adenocarcinoma. PET scan confirmed no evidence of disease, no further medical treatment was planned, and re-evaluation was performed 3 months after.

**Figure 4:**
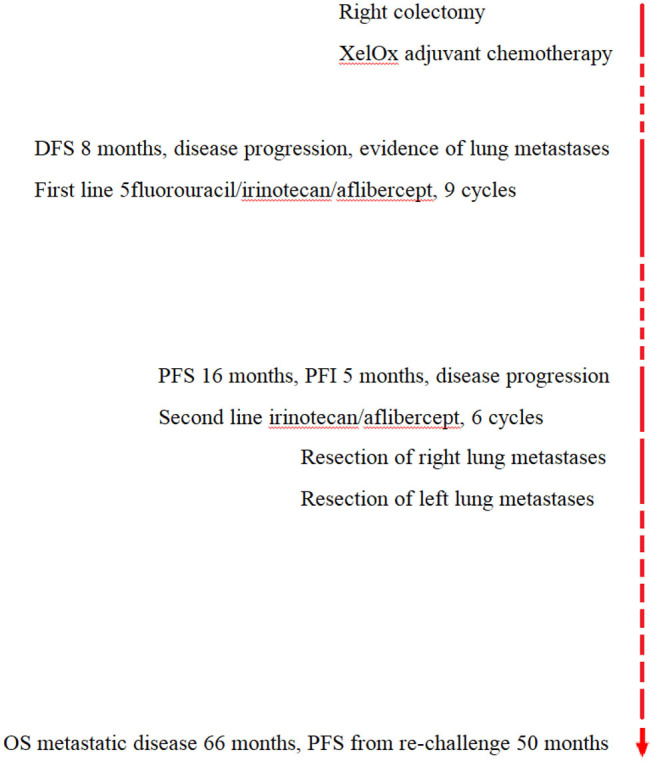
Timeline.

To date, clinical outcome shows PFS 50 months from aflibercept/irinotecan re-challenge, PFI 40 months after two-stage lung metastasectomies, and OS of metastatic disease 66 months, with no evidence of disease (Figure 4).

The procedures followed were in accordance with the ethical standards. Written informed consent was provided by the patient for proposed medical and surgical treatments, and to represent his clinical case. Written informed consent was obtained from the patient for the publication of any potentially identifiable images or data included in this article. Clinical management was shared with the patient, balancing oncological indication with patient's priorities, specifically regarding different available first-line treatment options, safety evaluation and implication on daily living, treatment modulation and interruptions caused by LTS, re-introduction, and integration with lung metastasectomies, followed by follow-up.

## Discussion

The case reported a yE patient with *KRAS* c.35 G>T (G12V) mutant MCRC rapidly progressing to adjuvant chemotherapy with bilateral lung metastases, unfit for intensive medical treatment owing to comorbidities, who was treated by first-line and re-introduction of aflibercept-containing chemotherapy followed by two-stage lung metastasectomies and gained in clinical practice by multidisciplinary treatment strategy long-term OS 66 months of metastatic disease with no evidence of disease at PFI 40 months.

Primary right-sided colonic adenocarcinoma with 5% mucinous component, Dukes C stage, harbored the second most prevalent (22.5%) *KRAS* c.35 G>T (G12V) mutation (15) and showed DFS 10 months, DFI 4 months after XelOx adjuvant chemotherapy. Prevalently occurring codon 12 *KRAS* mutations confer worse clinical behavior of CRC, and *KRAS* c.35 G>T mutation was an independent factor related with increased risk of recurrence and death (16), with significantly unfavorable DFS and OS in patients affected by Dukes C stage CRC (17); the poorer prognosis was not confirmed in other studies (18, 19). *KRAS* codon 12 mutations, specifically c.35 G>T, were related with worse OS compared with *KRAS*/*BRAF* wild-type cancers (20).

Effectiveness of intensive medical treatment integrated with radical resection of metastases raised clinical outcome of MCRC. In fit patients with MCRC, first-line FIr-B/FOx, developed from doublet and triplet schedules backbone (14, 21), reached ORR 82%, correlated with 26% secondary liver resections, PFS 12 months, OS 28 months (1); the prevalent codon 12 *KRAS* c.35 G>A mutant status was significantly associated with worse clinical outcomes of patients with MCRC treated with FIr-B/FOx compared with *KRAS/BRAF* wild-type and other *KRAS* mutant patients (22–24). FIr-B/FOx treatment integrated with metastasectomies significantly improved outcomes in liver-limited (PFS 17 months, OS 44 months) vs. O/MM patients (3, 22). Clinical outcome was not significantly affected by *KRAS* exon 2 (22), nor *KRAS*/*NRAS*/*BRAF* genotype, even if trendly favorable in triple wild-type (5). *KRAS* exon 2 wild-type liver-limited patients gained significantly favorable outcome because of secondary surgery, with respect to mutant (22). Increased efficacy of intensive first-line treatment and improved liver resection rate was confirmed in non-elderly *RAS*/*BRAF* wild-type patients treated with FIr-C/FOx-C triplet chemotherapy plus cetuximab, highly active and tolerable, reaching PFS 12 months (5).

The reported yE patient with secondary CIRS stage required tailored medical treatment (9, 11). Elderly status, PS ≥ 2, and/or comorbidities represent major parameters justifying treatment modulation to avoid limiting toxicities, preserve adequate quality of life, and to maintain proper dose intensities for expected activity. Elderly patients with MCRC are prevalent, and proper selection between intensive vs. tailored treatments is challenging, weighing expected tolerability and clinical outcome. Consecutive patients unsuitable for first-line intensive regimens, as a result of elderly (≥65 years) and/or comorbidity status, were 56%: elderly 76%, old-elderly 54%, PS 1–2 59%, intermediate/secondary CIRS stage 89%, with O/MM extension 79% (9). They were prevalently treated with modulated triplet or doublet regimens (49 and 40%, respectively). Patients treated with doublet regimens showed worse clinical outcomes (9). Unfit patients who underwent secondary liver surgery did not experience increased morbidity/mortality, reported to be significantly higher in elderly (8%) (25). Moreover, *KRAS* wild-type compared with mutant patients showed significantly favorable PFS, but not OS (22). *KRAS* c.35 G >A mutant genotype correlated with significantly worse PFS and OS vs. wild-type and/or other mutant (23, 24).

In patients with MCRC resistant to or progressing after oxaliplatin-based first-line chemotherapy, aflibercept addition to FOLFIRI significantly improved outcome (OS 13.5 months, PFS 6.9 months, ORR 19.8%) (6). Prolonged OS benefit was demonstrated: 38.5% at 18 months, 28.0% at 24 months, and 22.3 at 30 months (26), consistent across pre-specified randomization factors (27), only trendly favorable in *RAS/BRAF* wild-type and not according to sidedness (28). Adjuvant fast relapsers showed poorer efficacy and no survival benefit from addition of aflibercept (10.4 vs. 9.6 months) (7, 8); in Spanish real-life experience, PFS was 5.3–6.8 months (29, 30) and OS 12 months (30). Patients with metachronous vs. synchronous disease had significantly longer PFS 11 vs. 5 months, OS 17 vs. 10 months; left- vs. right-sided tumors had longer PFS 7 vs. 3 months, OS 12 vs. 8 months (30). Our clinical practice experience underlines the potential relevance of first-line aflibercept-based chemotherapy in a yE patient, unfit, *KRAS* mutant, with rapidly progressing MCRC achieving PFS 16 months, PFI 5 months. More re-challenge of the same schedule achieved a PR and PFS 7 months before lung metastasectomies, even at reduced aflibercept and irinotecan doses, and after fluorouracil discontinuation. Aflibercept vs. bevacizumab added to mFOLFOX6 reported equivalent median PFS 8.48 months and ORR 49.1 vs. 45.9%; *RAS*/*BRAF* mutations did not significantly correlate with PFS (31, 32). Aflibercept addition to mFOLFOX for six cycles, followed by maintenance and oxaliplatin reintroduction at progression, gained PFS 9.3 months (33). Aflibercept/FOLFIRI reached ORR 61.3% and PFS 8.4 months (34). Aflibercept/FOLFIRI for 12 cycles followed by aflibercept maintenance gained ORR 46.6%, PFS 8.4 months, and OS 20.9 months (35). At progression, first-line aflibercept-based chemotherapy was resumed, owing to long first-line PFS, consistent OR, and chemotherapy-free interval 5 months. After progression to first-line FIr-B/FOx, the outcome was significantly favorable in patients re-challenged with intensive regimen, unfavorable in c.35 G>A *KRAS* mutant (36). Triplet chemotherapy plus targeted agent re-challenge was offered to patients with previous OR, long PFS (≥10 months), off-treatment interval ≥3 months, no previous LT, and gained ORR 80%, related with 40% subsequent resections, PFS 13 months, and 2-years OS 80% (36).

Then, because of long-term control of bilateral lung metastastes during first-line aflibercept/irinotecan and re-challenge, bilateral resections of lung metastases were performed. The diagnosis of mucinous lung metastases may justify such a long OS 66 months of metastatic disease without evidence of disease at PFS 50 months and PFI 40 months from second stage lung metastasectomies, thus realizing the effectiveness of the integrated lung metastasectomies. In a retrospective Spanish real-life analysis of 32 patients who underwent surgical resection after aflibercept/FOLFIRI (37), PR was 56.3%, CR 3.1%, and resection rates R0 75.0%, R1 15.6%, and R2 9.4%. Secondary resection of different metastatic sites was performed: liver, 46.9%; lung, 25.0%; cytoreductive surgery for carcinomatosis, 15.6%; supra-adrenalectomy, 3.1%; liver and peritoneal carcinomatosis, 9.4%. Median PFS from surgery was 8.0 months and OS 37.3 months; in 22% of patients, aflibercept was resumed after surgery.

Reported yE, unfit patient underwent first-line MCRC treatment with reduced doses of aflibercept, irinotecan, and fluorouracil. Nevertheless, safety profile was characterized by LTS-ms, particularly G2 HFS lasting >2 weeks with different other G2–G1 toxicities. Because of limiting HFS and multiple pharmacogenomic alterations, 5-fluorouracil was discontinued; then, LTS-ms was observed, characterized by G3 hypertension, and re-introduction was planned with further aflibercept dose reduction. Reported prevalent toxicities with FOLFIRI/aflibercept were diarrhea (19.3%), mucositis (13.7%), asthenia (16.9%), HFS (2.8%), hypertension (2.9%), arterial (1.8%) and venous (7.9%) thromboembolic events, neutropenia (36.7%), and thrombocytopenia (3.3%) (8), the majority occurring within the first four cycles (26). In Spanish real-life experience, prevalent LT were neutropenia (7.9–15%), diarrhea (4.5–6.4%), asthenia (6.8–10%), and hypertension (3.4–6.8%) (30, 38, 39). Hypertension on-treatment was reported as a potential surrogate efficacy marker, associated with increased PFS 10.6 months and OS 17 months (30). In real-world data, >50% of patients requiring modified FOLFIRI schedules and doses had slightly older median age (63 years, range 35–82), 44% <65 years; no significantly different outcomes were reported according to modified schedules and doses, nor in elderly patients (39); G3–4 adverse events (40) and serious toxicity-related hospitalization were more common in elderly patients (≥65 years) (39). In Aflibercept Safety and Quality of Life Programs (41, 42), including an Italian experience with 43% yE, 10% early relapsers, 13.5 and 12% receiving, respectively, 5-fluorouracil and irinotecan lower dose, prevalent G3–4 toxicities were hypertension 24.1–28%, neutropenia 23.1–27.5%, and diarrhea 15.3–17%; no QoL worsening was reported; in elderly patients, G3–4 toxicities were lower than in VELOUR trial (81.3 vs. 89.3%) (41). As first-line treatment, aflibercept added to mFOLFOX6 reported G3–4 neutropenia 36.1%, hypertension 35.3%, proteinuria 9.2%, deep vein thrombosis 5.9%, and pulmonary embolism 5.9% (31). As we have previously reported in patients with MCRC treated by intensive first-line FIr-B/FOx and confirmed in the reported patient, toxicity induced by cancer medical treatments relies on individual clinical scenario of toxicity syndromes (TS), eventually LTS, frequently including multiple sites (LTS-ms) with clinical signs and symptoms of different degrees, requiring proper clinical management and drug modulations (1, 2, 12, 43). We introduced the innovative concept of LTS and defined LTS single site (LTS-ss), characterized by the LT alone, and LTS-ms, characterized by ≥2 LTs or a LT plus other, at least G2, non-LTs (1, 12, 13). Thus, LTS depicts toxicity burden in the individual patients. Cumulative G3–4 toxicities reported with FIr-B/FOx were equivalent in yE patients (≥65– <75 years), carefully selected by favorable PS, functional, comorbidity status: 44% overall; 46% in yE, mainly including diarrhea (69.2%), with significantly higher rate of LTS-ms vs. LTS-ss, compared with non-elderly (1, 12, 13). Overall, FIr-C/FOx-C induced LTS 65.5%, significantly more represented by LTS-ms (59%) vs. LTS-ss, prevalently LT plus other at least G2 non-limiting toxicities (34%) or ≥2 LTs (24%) (5). We previously showed that LTS monitoring could enhance evaluation of individual safety profile also in other different cancer settings (44–46).

Furthermore, the yE, unfit patient reported LTS-ms with XelOx adjuvant chemotherapy, confirmed all along with aflibercept-containing first-line chemotherapy and re-challenge. Fluorouracil and irinotecan doses were adjusted according to pharmacogenomic analyses, revealing multiple alterations of fluoropyrimidine and irinotecan metabolism, specifically severe 5-FUDR deficiency, SNPs of UGT1A1^*^28 homozygote 7 repeats, *ABCB1* c.C3435T and C1236T heterozygote, *MTHFR*—c.C667T homozygote, *DPYD* c.A166G heterozygote, and *TSER* 28bp heterozygote VNTR 2R/3R. Dihydropyrimidine dehydrogenase gene (*DPYD*) and *UGT1A1* SNPs variably influence fluoropyrimidines and irinotecan tolerability (47). In phase II trials evaluating triplet capecitabine, oxaliplatin, irinotecan, plus bevacizumab, or cetuximab, most relevant G3–4 toxicities were, respectively diarrhea (19 and 46%), neutropenia (3 and 7%), and asthenia (0 and 7%) (40). Limiting toxicity and treatment modulations were independently and significantly associated with *DPYD* c.496A > G (*P* = 0.022) and c.1896 T > C (*P* = 0.027), trendly with UGT1A1^*^28 SNPs (*P* = 0.054) (48). UGT1A1^*^28 allele determines decreased glucuronidation of SN-38 metabolite and enhances the risk of limiting irinotecan-related neutropenia (47–49). 5-FUDR was reported as a potential predictive biomarker of fluorophyrimidin toxicity in gastrointestinal cancers (50, 51). To further relate individual LTS occurrence, we performed specific companion analysis of pharmacogenomic biomarkers of fluorouracil and irinotecan safety profile 5-FUDR, *ABCB1, UGT1A1, CYP3A4*, and *DYPD* SNPs in 14 patients treated with FIr-C/FOx-C (5). Pharmacogenomic alterations involved 5-FUDR (43%), SNPs of *UGT1A1* (50%), *ABCB1* (71%), *CYP3A4* (14%), and *DYPD* (15%), in the reported range (47, 49). Most patients (65%), specifically those who developed gastrointestinal LTS (78%), showed >1 pharmacogenomic alteration, including reduced FUDR, *UGT1A1*, or *CYP3A4* SNPs (range 1–3), thus predicting occurrence of LTS-ms in patients at risk of gastrointestinal LT (5). Thus, apart from the primary evaluation of patient as unfit, owing to yE and comorbidities, LTS reported with different chemotherapy combinations and related to detected pharmacogenomic alterations further confirms how complex, careful, and rigorous it would be in clinical practice to monitor medical treatments of individual patients with cancer.

## Conclusion

In clinical practice, a complex management evaluating patient-related clinical parameters and *RAS*/*BRAF* genotype of patients with MCRC, particularly the elderly and/or those who are unfit because of comorbidities, is required to properly tailor multidisciplinary medical and surgical treatment strategies to gain optimal activity and long-term efficacy, integrated with careful monitoring of toxicity syndromes, potentially related to pharmacogenomic alterations.

## Ethics Statement

Written informed consent was obtained from the patient for the publication of any potentially identifiable images or data included in this article.

## Author Contributions

GB and ER were the medical oncologists responsible for clinical and bioclinical management of patient. AD'A, SG, and EAR were abdominal and thoracic surgeons responsible for surgical management of patient. MS performed pharmacogenomic analyses. All authors read and approved the final manuscript. All authors contributed to the article and approved the submitted version.

## Conflict of Interest

The authors declare that the research was conducted in the absence of any commercial or financial relationships that could be construed as a potential conflict of interest. The reviewer AB declared a past co-authorship with one of the authors MS to the handling editor.
